# An Informatics-Based, Payer-Led, Low-Intensity Multichannel Educational Campaign Designed to Decrease Postdischarge Utilization for Medicare Advantage Members: Retrospective Evaluation

**DOI:** 10.2196/63841

**Published:** 2025-05-27

**Authors:** Danica Fernandes, Elise Kokonas, Jai Bansal, Ken Hayashima, Brian Hurley, Annabel Ryu, Snehal Mhatre, Mohammed Ghori, Kelly Jean Craig, Amanda L Zaleski, Lily Vogel, Alena Baquet-Simpson, Daniel Reif

**Affiliations:** 1 Analytics & Behavior Change, Aetna, CVS Health, New York, NY, United States; 2 Marketing, Aetna, CVS Health, New York, NY, United States; 3 Clinical Evidence Development, Medical Affairs, CVS Health, Corporate Office, Wellesley, MA, 05401, United States, 1 802-489-8816; 4 Medical Affairs, Aetna, CVS Health, Wellesley, MA, United States

**Keywords:** clinical informatics, digital health, health behavior, hospital readmission, personalized education, population health, human-centered design, design, human centered, educational, medicare, behavior change, outreach campaign, readmission, inpatient, messaging campaign, messaging, utilize, utilization

## Abstract

**Background:**

Readmission avoidance initiatives have been a priority for the Centers for Medicare & Medicaid Services for over a decade; however, interventions are often high-intensity, costly, and resource-intensive, and therefore, rarely scalable or sustainable. Large national payers are in a unique position to leverage data to identify members in real-time who are at high risk of readmission to prioritize the scaled delivery of tailored behavior change techniques to provide an educational intervention to modify health behaviors.

**Objective:**

This study aims to examine the impact of an informatics-driven, multichannel educational messaging campaign implemented to decrease 30- and 90-day acute inpatient readmissions and emergency department (ED) visits among Medicare Advantage members of a large national payer.

**Methods:**

A quality improvement initiative was designed and implemented to provide an evidence-based outreach campaign using human-centered design and behavior change principles to deliver multiple intervention functions, including timely, contextual, and relevant delivery of education, enablement, and persuasion, to reinforce health-promoting behaviors related to planned or unplanned inpatient admissions. Outcomes, including 30- and 90-day acute inpatient readmissions and ED visits, were retrospectively evaluated from Medicare Advantage members enrolled in a large national health plan residing across the United States between May 2020 and July 2022. Leveraging utilization management data, rules-based logic identified members (N=368,393) with a planned acute inpatient procedure (ie, preadmission) or discharged from an acute hospital stay (ie, postdischarge) within 15 days. Members were sequentially assigned to a standard (N=141,223) or an enhanced (N=227,470) messaging group, whereby the standard group received usual outreach and the enhanced group received an educational intervention via a messaging campaign deployed through multiple low-intensity communication channels (eg, text message, email, direct mail) in addition to standard outreach.

**Results:**

Members who received enhanced outreach had fewer relative 30-day acute inpatient readmissions (−4.1%, 95% CI −5.5% to −2.7%; *P*<.001) and ED visits (−3.4%, 95% CI −5.0% to −1.7%; *P*<.001) compared with members receiving standard outreach. Similarly, these findings persisted for relative 90-day outcomes such that members receiving enhanced outreach experienced fewer acute inpatient readmissions (−5.4%, 95% CI −6.5% to −4.3%; *P*<.001) and ED visits (−3.8%, 95% CI −5.0% to −2.5%; *P*<.001) compared with members receiving standard outreach messaging.

**Conclusions:**

Behavior change techniques deployed via educational interventions as low-intensity multi-channel outreach is an effective strategy to reduce avoidable 30- and 90-day inpatient readmissions and ED visits in recently discharged Medicare Advantage members (primarily >65 years).

## Introduction

Acute inpatient readmissions pose a significant challenge to the US health care system, affecting patient outcomes, health care costs, and overall quality of care. Despite increasing health system focus on readmission avoidance initiatives, hospital readmissions remain a major burden for health care organizations, payers, and patients. On average, there are ≈3.8 million readmissions per year, contributing to US $452.4 billion in US health care costs, of which Medicare members account for ≈60% [1]. As such, the Centers for Medicare & Medicaid Services introduced the Hospital Readmission Reduction Program (HRRP) in 2012 with the primary goal of addressing excessive hospital readmissions and improving the quality of health care delivery [2]. Key activities of the HRRP include identifying high-risk patients, comprehensive discharge planning, patient education, medication reconciliation, timely follow-up appointments, care coordination with primary care providers, and proactive outreach to patients postdischarge to monitor their health status and address any concerns. Under the HRRP, the Centers for Medicare & Medicaid Services imposes financial penalties on hospitals with higher-than-expected 30-day readmission rates for certain conditions, including, but not limited, to acute myocardial infarction, heart failure, and pneumonia [2].

Now, 10+ years since its inception, there has been a dearth of literature focused on reducing hospital readmissions. Of the existing studies, a majority focus on high-intensity interventions that are costly and resource-intensive, such as home visits and clinician-led telephonic outreach to deliver high-touch member education, discharge planning, care coordination, and transitions of care [3-6]. While such interventions have shown great promise, they are generally not universally sustainable for large-scale implementation. In addition, many readmission avoidance programs primarily focus on specific high-risk groups, leaving a significant portion of the patient population underserved. Thus, there is an unmet need to identify practical and cost-effective strategies that can be operationalized and delivered at scale.

Large national payers hold a unique position in the health care landscape owing to continuous data ingestion of claims and clinical informatics data. Leveraging this rich and diverse data foundation, payers are well-poised to identify members in real-time who are at high risk of readmission to prioritize the frequency and intensity of behavior change techniques and interventions. Combined with clinical expertise and other foundational capabilities (ie, multi-channel tools, interoperability, and plan benefit design), these data-informed insights have great potential to enable the delivery of low-cost interventions to modify members’ health behaviors associated with a planned inpatient procedure or following discharge from an acute hospital stay. These tailored intervention functions complement and extend existing initiatives across the health care ecosystem, enhance the patient and member care experience, and effectively optimize the use of health care resources on a population level.

As such, the purpose of this study was to explore the impact of a payer-led quality improvement initiative that used behavior change techniques to deliver low-intensity interventions, primarily an education-based campaign with persuasion and enablement, as an innovative approach to address the persistent challenge of hospital readmissions and related health care use outcomes. Specifically, it was hypothesized that Medicare Advantage members receiving enhanced outreach using a multichannel educational campaign would exhibit lower relative 30- and 90-day acute inpatient readmissions and emergency department (ED) visits compared with members receiving standard of care messaging (ie, usual outreach).

## Methods

### Study Overview

This study represents a retrospective evaluation of a quality improvement intervention. Briefly, a personalized, evidence-based, informatics-enabled campaign framework was designed and implemented with the overall goal to reduce avoidable hospital readmissions in the Medicare Advantage (primarily >65 years) population. Eligible members were sequentially assigned to a standard or enhanced messaging intervention consisting of evidence-based, multichannel lay education. Administrative claims data were deidentified, aggregated, and analyzed to compare relative changes in 30- and 90-day acute inpatient readmissions and ED visits in a group of Medicare Advantage members that received the enhanced messaging campaign compared with those who received the standard messaging campaign.

### Overview of the Campaign Framework

**Figure 1.**
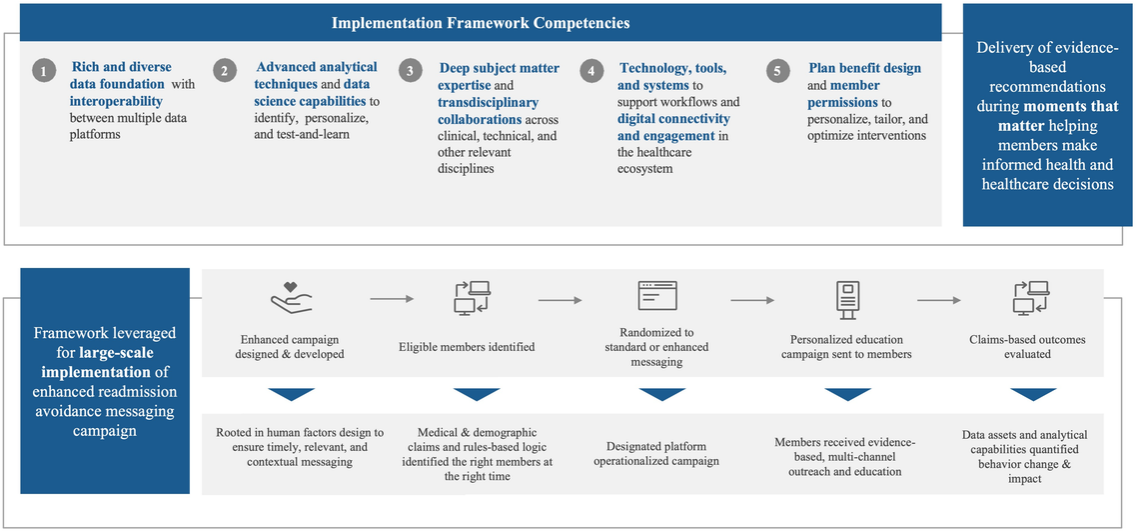
A high-level overview of the campaign framework. Key foundational competencies directly enable the identification, delivery, and evaluation of an informatics-based, readmission avoidance educational messaging campaign for Medicare members of a large national payer. Potential members are identified through rule-based logic that is highly dependent on a rich and diverse data foundation. Model sets are derived from a data warehouse that continuously ingests data from numerous internal platforms with interoperability. Advanced analytics identifies and routes eligible members to a dedicated platform to operationalize the delivery of evidence-based, multichannel outreach and education campaigns (standard or enhanced). Data assets and analytical capabilities facilitate the retrospective evaluation of behavior change and downstream impacts on health care use patterns.

A quality improvement initiative was designed and implemented to provide an evidence-based outreach campaign using behavior change techniques to deliver multiple intervention functions, including education, enablement, and persuasion, to modify health behaviors related to planned or unplanned inpatient admissions. An education-based outreach intervention was designed with the intention of supporting Medicare Advantage members in taking their “next best action” to better health through analytics-informed behavioral changes to modify health behavior before and after hospital admission. The campaign framework was operationalized across six key components (Figure 1): (1) a rich and diverse data foundation; (2) interoperability between multiple internal and external data platforms; (3) application of analytical techniques and data science capabilities, (4) a designated platform to serve as the centralized technology to operationalize campaigns; (5) deep subject matter expertise, and (6) connectivity and engagement in the health care ecosystem to deliver actionable recommendations for members, providers, or both.

### Member Identification

The campaign was deployed to Medicare Advantage members enrolled in a large national health plan with an acute inpatient index event and discharged to a home setting between May 2020 and July 2022. One index admission was associated with each member’s study eligibility, which occurs once per 180-day window. Members were excluded from study evaluation if they were discharged to a nonhome setting (eg, skilled nursing facilities, long-term care); enrolled in Dual-Eligible Special Needs Plans; had nonimpactable conditions (ie, trauma, pregnancy); or were admitted for maternity-related or behavioral health events.

### Standard Versus Enhanced Messaging Group Allocation

Existing technological capabilities were leveraged to operationalize campaigns. Specifically, a marketing technology application (previously developed in-house in 2018) served as the centralized tool to enable identification, risk stratification, randomization, channel delivery, engagement, and reporting. Briefly, the platform architecture integrates multiple internal, third-party, and other external data, systems, and services. Defined modules enable data input, processing, and campaign deployment. Precise member targeting and agile data science and marketing pods drive rapid and iterative test-and-learn experimentation approaches that support high-volume test cells to evaluate the impact of campaign-related interventions.

Leveraging these standardized processes, members were sequentially assigned to standard or enhanced messaging groups following response adaptive-randomization procedures with dynamic adjustment for covariate imbalance [7]. Briefly, the standard intervention consisted of usual outreach messaging (ie, single-channel, single-time point evidence-based educational messaging) to members with conditions that were high-risk for readmission while the enhanced intervention used member- and condition-informed personalization to augment the mode, frequency, timing and content of educational messaging in an expanded population (Table 1).

**Table 1.** Comparison of standard versus enhanced messaging intervention components.

Intervention component	Standard intervention	Enhanced intervention
Population	High-risk or select conditions	All eligible members with an acute inpatient index event
Data sources	Medical and administrative claims	Daily list of acute inpatient stays Precertification vendor data Provider information Plan benefit structure
Personalizationlogic	Member-centric content (see messaging content below)	Machine learning to optimize member’s channel preferences Just-in-time messaging Tailored content (see messaging content below)
Channel delivery	Channels informed by member permissions Variable; often single channel	Multichannel (eg, email, direct mail, SMS, and IVR^a^) approach Provider (PCP)^b^ fax notifications
Timing		
Preadmission	None	Within 15 d prior to admission
Postdischarge	High-risk or select conditions	3‐5 d after discharge to home
Recovery	High-risk or select conditions	7‐30 d after previous message
Frequency	One time	Multiple touchpoints
Messaging content	Clinically relevant (ie, condition-specific) Informed by evidence-based guidelines	Behavior change technique groupings (eg, goals and planning, shaping knowledge), and intervention functions (eg, education, persuasion, and enablement) Multi-component communication campaign (knowledge sharing, reminders, tracker tool, reinforcement of provider follow-up, complications awareness, etc) Rooted in human-centered design Condition-specific considerations Reinforcement of additional plan-enabled resources and support (eg, transportation)

a IVR: interactive voice response.

b PCP: primary care provider.

### Standard Intervention Description

The standard group received the usual outreach messaging. In general, usual member-facing communication leverages a combination of demographic information, health history, and behavioral economics to create personalized, evidence-based awareness and education campaigns. Personalized messaging campaigns are deployed through multichannel modes of communication delivery, including: digital (eg, SMS, multimedia message service, email, interactive voice response [IVR], 1:1 personalized websites or mobile applications); traditional (eg, direct mail, telephonic); or in-person (eg, provider-led, retail brick-and-mortar store) channels.

General principles of member-facing campaigns are to (1) serve as a trusted source of evidence-based, credibly sourced health education; (2) provide strategies to overcome barriers to enable decision-making; and (3) deliver personalized, relevant, and timely information that facilitates informed health care decisions. Education-based campaigns are grounded in well-established, evidence-based guidelines and best-practice recommendations for population health (eg, United States Preventive Services Task Force, Centers for Disease Control and Prevention, and American Heart Association). All educational content reinforces or references publicly available health education intended for lay audiences and approved by an internal panel of medical directors with subject matter expertise.

Primary outcomes of member-facing communications vary depending on the use case but are designed to favorably impact clinical outcomes, appropriate health care utilization, medical health care spend, quality metrics, or member satisfaction. For example, many messaging campaigns are designed to favorably augment outcomes related to primary (eg, vaccinations), secondary (eg, screenings), tertiary (eg, disease management), and quaternary (eg, prevent overmedicalization) care activities. Note that standard outreach communications may differ (ie, frequency, intensity, and type) based on member permissions, plan benefit design, gaps in care, and member risk/need. In the event of potentially redundant outreach initiatives, standard campaigns may be suppressed to facilitate test-and-learn outreach campaigns that are most likely to optimize member outcomes. Specific to this use case, examples of interventions that members may receive include payer-led readmission avoidance program support; postdischarge, telephonic-based, nurse care manager outreach; pharmacist-based medication reconciliation; home health services; remote monitoring; and social support services.

### Enhanced Intervention Description

The enhanced group received usual (ie, standard) outreach messaging in addition to personalized readmissions-focused educational outreach using a low-intensity multichannel approach. The enhanced campaign was designed and implemented by a transdisciplinary team composed of experts across data science, clinical, economic, behavioral, marketing, and public health disciplines. Human factors principles were integrated into an agile, rapid-cycle testing framework [8,9] to ensure usability, accessibility, and real-world applicability of readmission reduction interventions for the identified user and context; Medicare members with a recent or planned hospitalization. User requirements for the enhanced intervention were informed by best practices for readmission prevention [2], including recommended transitional care activities (ie, timely postdischarge provider follow-up) and self-monitoring and self-management activities (ie, medication adherence, symptom tracking, and postacute recovery). Human-centered design principles were then applied to enhance local adaptability based on anticipated needs, barriers, and facilitators that were informed by the existing literature [3,9-16] and in-house medical directors with subject matter expertise. These specifications informed the development of the final campaign that was pilot-tested against design requirements prior to large-scale implementation. Continuous feedback loops, including call transcripts, surveys, and evaluations, enabled iterative refinements to messaging, workflows, and intervention delivery, ensuring alignment with user needs and real-world constraints.

The overall goal of the campaign was to support, empower, and encourage members to engage in clinical actions aligned with guideline-directed care. Personalized, timely outreach at critical touchpoints in a member’s journey (ie, preadmission and postdischarge) ensured that messaging was contextually relevant and most likely to translate to goal-concordant behavior change related to planned or unplanned hospital stay. Messaging was grounded in behavioral economics of decision-making, specifically the “foot-in-the-door” technique [17], which facilitates engagement by first encouraging small, manageable tasks (eg, filling out a preadmission checklist or a postdischarge recovery tracker), which often translates to participation with a larger ask, such as avoiding unnecessary health care utilization [17]. Additionally, behavior change technique groupings (eg, goals and planning, shaping knowledge) [18], and intervention functions (eg, education, persuasion, and enablement) [19] were incorporated to reinforce health-promoting behaviors.

**Figure 2.**
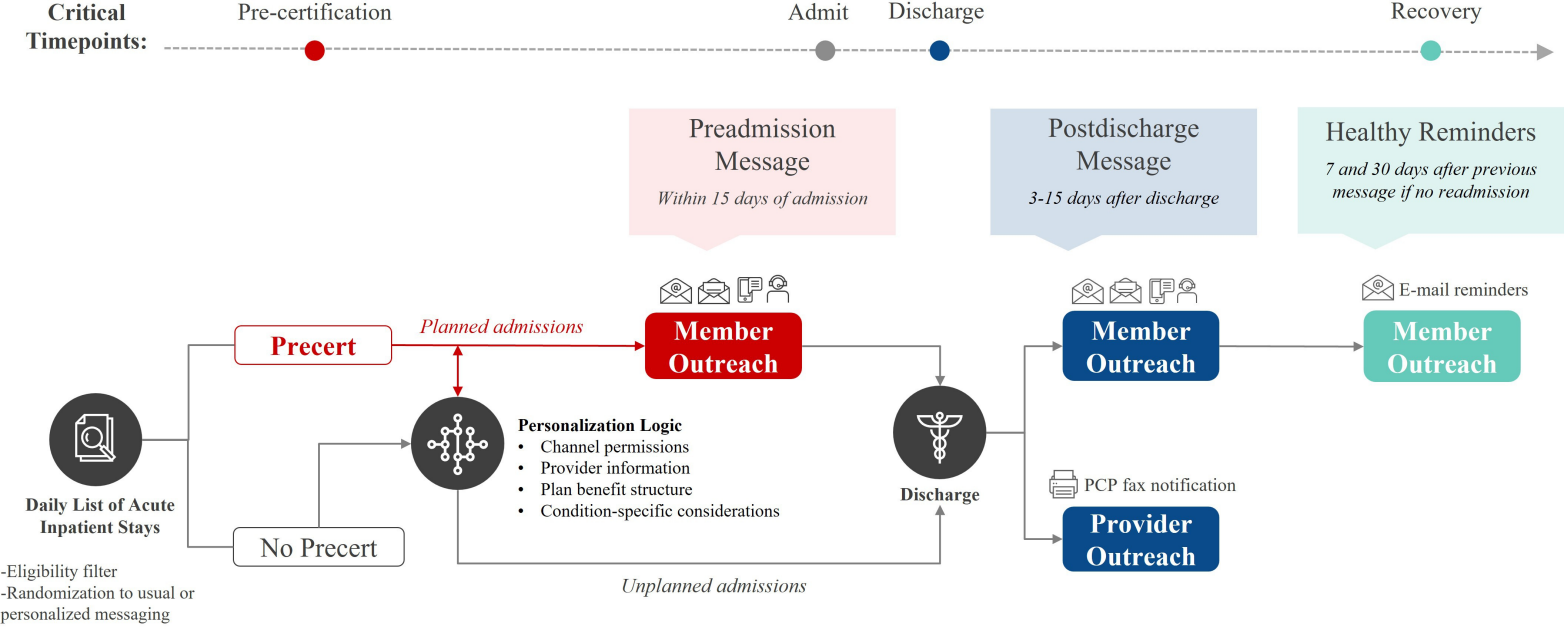
Readmissions campaign member flow. Overall campaign logic flow and cadence containing both preadmission (where applicable) and postdischarge messaging and channels. Potential channels for initial member outreach were informed by member permissions and included: e-mail, direct mail, SMS, and interactive voice response (IVR). Provider fax notifications were also sent to all members with a provider on file. PCP: primary care provider.

The campaign (Figure 2) identified members with a planned acute inpatient procedure (ie, preadmission) using the precertification process or those who were discharged from an acute hospital stay (ie, postdischarge), both within a 15-day event window time span. Just-in-time lay educational messaging was strategically timed to reinforce key recovery actions at critical touchpoints in the member’s journey. The outreach strategy used all available communication channels based on member preferences, ensuring inclusivity across digital and nondigital mediums. Preadmission outreach provided timely education on essential preparation steps, including instructions to (1) prepare the home environment, (2) schedule provider follow-up visits and manage prescription fills, and (3) stay on track with recovery plans. Postdischarge outreach supported recovery during the critical 30-day period by delivering tools and information. All members received a recovery tracker calendar with designated fields to record self-reported signs, symptoms, and notes, along with stickers to reinforce daily use. The recovery tracker was designed for portability, enabling members to easily bring to follow-up appointments to facilitate patient-provider communication. Messaging emphasized the importance of provider follow-up, medication adherence, and symptom recognition for complications that would warrant further care. In addition, contact information was provided to facilitate inbound telephonic outreach to assist with potential questions or needs.

Examples of enhanced member-facing preadmission and postdischarge messaging can be referenced in MultimediaAppendices 1  2, respectively. Human-centered design ensured messaging was accessible and engaging [11]. For example, plain language and large fonts optimized readability and usability, addressing common barriers to comprehension. To accommodate varying levels of digital literacy, all members received direct mail (at minimum), with reinforcement through digital channels (eg, SMS, email, IVR) based on member preferences, ensuring engagement was not limited by access or comfort with technology. Operational efficiencies were implemented to improve the timeliness of direct mail delivery. Creatives were preprinted and mailed daily to ensure arrival within 3 days of identification. Personalization was further enhanced to improve relevance and usefulness using rules-based logic informed by provider relationships, medical and social needs, and plan benefit structure. For example, for members with a provider of record, fax notifications were incorporated to reinforce patient-provider communication and care coordination. Where plan benefits allowed, postdischarge messaging included transportation options and additional support for medical and social needs.

### Campaign Evaluation

#### Data Sources and Study Setting

**Figure 3.**
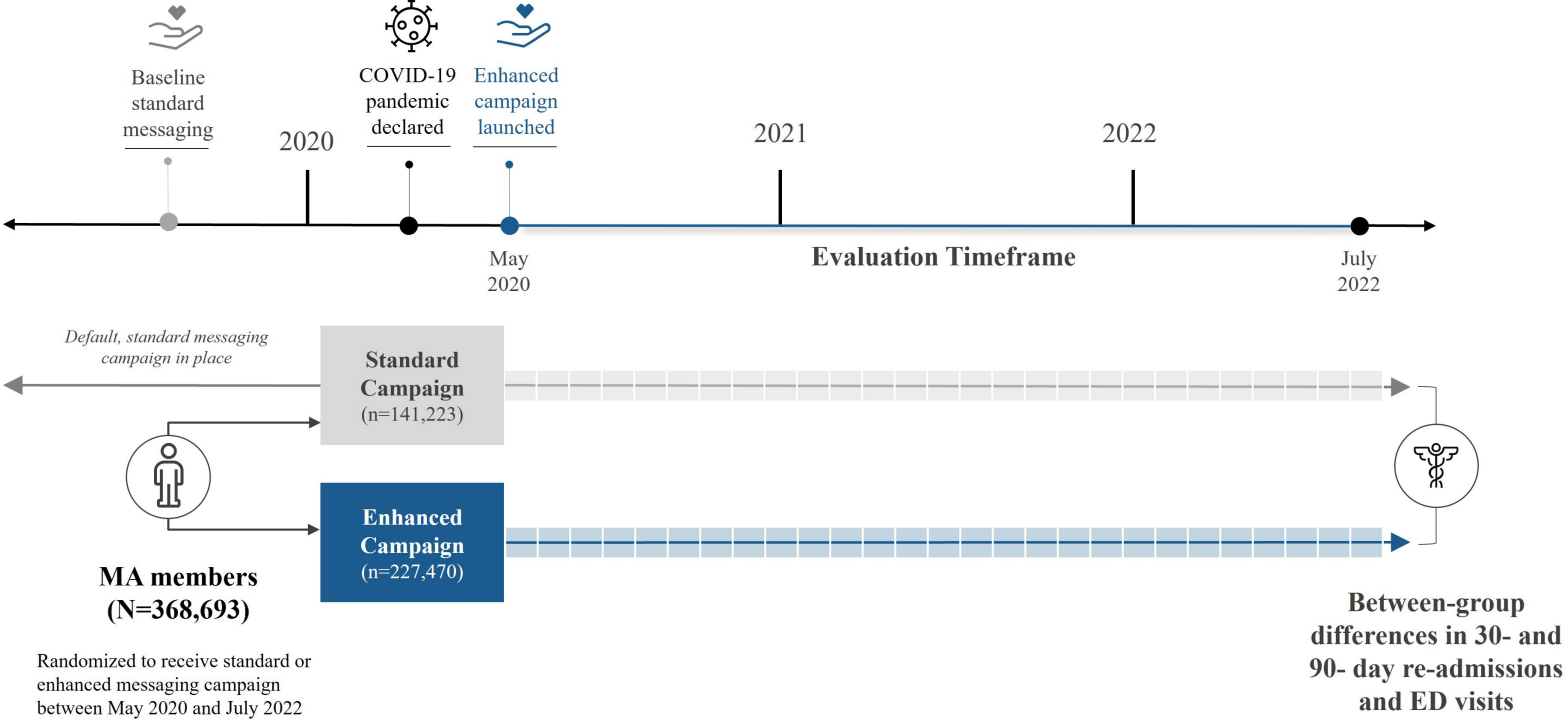
Study design overview. The campaign experimentation framework was leveraged to conduct a retrospective analysis of 30- and 90-day outcomes (ie, hospital readmissions and emergency department [ED] visits) between members who received the enhanced messaging campaign compared with a cohort of members who received the standard campaign.

Retrospective demographic and medical claims data were deidentified, aggregated, and analyzed to determine the impact of the enhanced versus standard educational campaign on primary outcomes for the evaluation timeframe (ie, May 2020 to July 2022). The study design overview is provided in Figure 3. Claims data included ED diagnoses, procedures, sites of care, and provider information. Additional data fields included aggregations of the above information in the forms of medical cases, episode treatment groups, and chronic condition flags. Demographic information collected during health insurance enrollment included self-reported sex, age, plan benefit design, location, and census tract statistics.

#### Inclusion and Exclusion Criteria

All participants were enrollees of a Medicare Advantage health plan provided by a large national payer. Inclusion criteria included: (1) receipt of ≥1 standard or enhanced messaging campaign intervention throughout the study evaluation period. Potential participants were excluded if they: (1) did not meet inclusion criteria or (2) were members of a commercial, dual coverage, or indemnity insurance plan.

### Ethical Considerations

The Sterling Institutional Review Board reviewed and approved the study (#10132) as an exempt study under 45 CFR 46.104(d)(4). Informed consent was not obtained, as an exemption determination was provided. In addition, a waiver of Health Insurance Portability and Accountability Act authorization for the use and disclosure of aggregated, deidentified member data was obtained. No compensation was provided.

### Statistical Analyses

Two-tailed unpaired *t* tests examined between-group differences in baseline characteristics to ensure adaptive randomization techniques effectively achieved balance. Change in acute inpatient readmission rate was calculated as the difference in the average number of readmissions per 100 members (ie, readmission rate) between the standard and enhanced outreach groups. The relative reduction in inpatient readmissions was calculated as the between-group difference in readmission rates divided by the standard outreach group’s readmission rate and expressed as a percentage ±95% CI. Change in ED visits was calculated as the difference in the average number of ED visits per 100 members between the standard and enhanced outreach groups. The relative decrease in ED visits was calculated as the between-group difference in ED visits divided by the standard outreach group’s ED visit rate and expressed as a percentage ±95% CI.

Secondary analyses explored differences in 30- and 90-day acute inpatient readmissions and ED visits by age brackets (ie,<65 yr, 65‐69 yr, 70‐74 yr, 75‐79 yr, 80‐84 yr, and >80 yr), geographic region (ie, Midwest, Northeast, Southeast, West), geographic area (ie, urban, suburban, rural), as well as common comorbidities (eg, dementia, diabetes mellitus, low back pain, and obesity).

Covariate balance was confirmed by assessing standardized mean differences for all variables reported. Chi-square tests were used to test the statistical significance of the outreach impact on primary outcomes. Two-tailed unpaired *t* tests were used to explore between-group differences, with statistical significance defined as *P*<.05.

## Results

### Baseline Characteristics

Table 2 details the baseline characteristics of the standard (N=141,223) and enhanced (N=227,470) outreach groups. On average, the total included study population (N=368,693) was comprised of older adult (mean age:75.3 yr) males (n=181,083 [49.1%]) and females (n=187,610 [50.9%]); a majority of which residing in rural geographies (n=189,594 [51.4%]) with top 3 comorbidities being heart conditions (n=153,353 [41.6%]), diabetes mellitus type 1 or 2 (n=115,432 [31.3%]), and low back pain (n=114,954 [31.2%]). There were no between-group differences in baseline demographic characteristics, risk factors, or comorbidities (all *P*>.12). Of note, mean age was marginally higher in the enhanced (75.4 yr) versus standard (75.3 yr) group (*P<*.001), though this is not considered clinically significant.

**Table 2.** Baseline demographic and clinical characteristics among the total study population and comparison by group.

Characteristic	Total population (N=368,693)	Standard outreach (n=141,223)	Enhanced outreach (n=227,470)	SMD^a^	*P* value
Sex, n (%)				0.005	.12
Male	181,083 (49.1)	69,623 (49.1)	111,460 (49.0)		
Female	187,610 (50.9)	71,600 (50.7)	116,010 (51.0)		
Age (years), mean (SD)	75.3 (9.9)	75.4 (9.9)	75.3 (10.0)	–0.010	<.001
Age at admission (years), n (%)					
<65	37,889 (10.3)	14,687 (10.4)	23,202 (10.2)	–0.005	.91
65‐69	56,269 (15.3)	21,466 (15.2)	34,803 (15.3)	0.002	.89
70‐74	76,578 (20.8)	28,809 (20.4)	47,769 (21.0)	0.012	.89
75‐79	75,245 (20.4)	28,386 (20.1)	46,859 (20.6)	0.012	.88
80‐84	58,677 (15.9)	22,737 (16.1)	35,940 (15.8)	–0.006	.88
>85	64,035 (17.5)	25,138 (17.9)	38,897 (17.2)	–0.016	.87
US region, n (%)					
Midwest	102,952 (27.9)	39,260 (27.8)	69,518 (28.0)	0.003	.85
Northeast	124,446 (33.8)	48,016 (34.0)	83,413 (33.6)	–0.007	.84
Southeast	92,401 (25.1)	35,306 (25.0)	62,369 (25.1)	0.001	.86
West	48,894 (13.2)	18,641 (13.1)	32,705 (13.2)	0.004	.90
Geographic area, n (%)					
Urban	90,950 (24.7)	35,447 (25.1)	55,503 (24.4)	–0.015	.85
Suburban	88,149 (23.9)	33,329 (23.6)	54,820 (24.1)	0.010	.87
Rural	189,594 (51.4)	72,447 (51.2)	117,147 (51.5)	0.005	.80
Comorbidities, n (%)					
Behavioral health^b^	96,488 (26.2)	36,436 (25.8)	60,052 (26.4)	0.013	.86
Cancer	89,059 (24.2)	33,329 (23.6)	55,730 (24.5)	0.021	.87
COVID-19	12,308 (3.3)	4802 (3.4)	7507 (3.3)	0.000	.95
Dementia	21,298 (5.8)	8332 (5.9)	12,966 (5.7)	–0.008	.93
Diabetes mellitus	115,432 (31.3)	43,779 (31.0)	71,653 (31.5)	0.011	.85
Heart conditions^c^	153,353 (41.6)	58,043 (41.1)	95,310 (41.9)	0.017	.83
Hepatitis	5530 (1.5)	2118 (1.5)	3412 (1.5)	0.004	.97
HIV	1106 (0.3)	424 (0.3)	682 (0.3)	0.006	.99
Low back pain	114,954 (31.2)	43,073 (30.5)	71,881 (31.6)	0.024	.85
Multiple sclerosis	1843 (0.5)	706 (0.5)	1137 (0.5)	–0.004	.99
Obesity	82,336 (22.3)	30,928 (21.9)	51,408 (22.6)	0.017	.88
Parkinson disease	5162 (1.4)	1977 (1.4)	3185 (1.4)	0.003	.97
Rheumatoid arthritis	10,920 (3.0)	4095 (2.9)	6824 (3.0)	0.006	.95
Renal conditions^d^	71,385 (19.4)	27,256 (19.3)	44,129 (19.4)	0.006	.88
SUD^e^	20,506 (5.6)	7767 (5.5)	12,738 (5.6)	0.005	.94

a SMD: standardized mean difference.

b Anxiety, bipolar disorder, depression, psychoses, eating disorders, and postpartum disorders.

c Heart failure, congenital heart disease, and ischemic heart disease.

d End-stage renal disease and chronic renal failure.

e SUD: substance use disorder.

### Thirty-Day Inpatient Readmissions and ED Visits

Members in the enhanced outreach group experienced a 4.1% (95% CI 2.7%‐5.5%) relative reduction in acute inpatient readmissions compared with the standard outreach group (*P*<.001). In addition, the enhanced outreach group experienced a 3.4% (95% CI 1.7%‐5.0%) relative reduction in unplanned ED visits compared with the standard outreach group (*P*<.001).

Secondary analyses (Table 3) revealed that members between the ages of 70 and 74 years experienced the greatest relative decrease (5.8%) in acute inpatient readmissions, followed by members who were 65‐69 yr (5.3%), and 75‐79 yr (4.5%) (all *P*<.05). Members who reside in the Northeast (8.0%) and Midwest (3.4%) experienced a greater relative reduction in acute inpatient readmissions (both *P*<.05) compared with those in the Southeast or West, as did those in suburban (7.6%) compared with members in urban (3.8%) and rural areas (both *P*<.05). Certain comorbidities also demonstrated a relationship with decreased frequency of 30-day readmissions (Table 2), including behavioral health, cancer, diabetes mellitus type 1 or 2, and heart conditions (all *P*<.05).

**Table 3.** Relative change in 30-day acute inpatient readmissions and emergency department (ED) visits by demographic and clinical characteristics.

Characteristic	Change in 30-day inpatient readmissions (%), (95% CI)	*P* value	Change in 30-dayED visits (%), (95% CI)	*P* value
Age at admission (years)				
<65	−2.3 (−5.7 to 1.2)	.38	−1.1 (–4.8 to 2.6)	.57
65‐69	−5.3 (−8.5 to −2.1)	.02	−1.9 (–5.5 to 1.7)	.36
70‐74	−5.8 (−8.6 to −2.9)	.005	−5.7 (–9.1 to −2.4)	.001
75‐79	−4.5 (−7.2 to −1.8)	.02	−6.7 (–10.0 to −3.4)	<.001
80‐84	−4.1 (−7.1 to −1.1)	.06	1.9 (−1.5 to 5.2)	.36
>85	−1.4 (−4.1 to 1.3)	.51	−4.0 (−7.3 to −0.7)	.03
Region				
Midwest	−3.4 (−5.7 to −1.1)	.04	−2.8 (−5.3 to −0.2)	.04
Northeast	−8.0 (−10.2 to −5.8)	<.001	−5.7 (−8.3 to −3.2)	<.001
Southeast	−0.1 (−2.4 to 2.2)	.95	−1.8 (−4.6 to 1.1)	.28
West	−2.4 (–5.7 to 0.8)	.34	−2.0 (−5.7 to 1.6)	.34
Geographic area				
Urban	−3.8 (−6.1 to −1.4)	.03	–3.8 (–6.9 to –0.7)	.02
Suburban	−7.6 (−10.2 to −5.1)	<.001	–6.8 (–9.8 to –3.7)	<.001
Rural	−2.3 (−4.0 to −0.6)	.07	–1.9 (–3.7 to –0.1)	.07
Comorbidities				
Behavioral health^a^	−3.4 (−5.6 to −1.1)	.049	–2.7 (–5.2 to –0.1)	.05
Cancer	−4.2 (−6.5 to −1.9)	.02	−5.4 (−8.3 to −2.5)	<.001
COVID-19	−0.3 (−6.0 to 5.5)	.95	−8.4 (−15.5 to −1.3)	.008
Dementia	−0.7 (−5.2 to 3.8)	.85	−5.8 (−11.5 to −0.2)	.04
Diabetes mellitus	−4.4 (−6.4 to −2.4)	.003	–4.6 (–7.0 to –2.2)	<.001
Heart conditions ^b^	–3.1 (–4.8 to –1.4)	.02	−2.4 (−4.5 to −0.4)	.04
Hepatitis	−5.8 (−14.1 to 2.4)	.31	−21.6 (−34.8 to −8.3)	<.001
HIV	−5.5 (−26.0 to 15.0)	.70	−30.1 (−60.3 to 0.1)	.005
Low back pain	−3.0 (−5.2 to −0.9)	.06	−5.8 (−8.3 to −3.3)	<.001
Multiple sclerosis	−2.8 (−19.8 to 14.2)	.83	1.8 (−17.3 to 20.9)	.86
Obesity	−2.7 (−5.2 to −0.3)	.15	−2.1 (−4.9 to 0.8)	.21
Parkinson disease	−5.7 (−15.3 to 3.9)	.42	−16.5 (−29.3 to −3.7)	.001
Rheumatoid arthritis	−7.2 (−14.0 to −0.3)	.14	−10.0 (−18.1 to −1.9)	.02
Renal conditions^c^	−1.7 (−4.0 to 0.6)	.33	0.9 (−2.0 to 3.7)	.59
SUD^d^	−3.8 (−8.2 to 0.7)	.25	1.1 (−4.0 to 6.2)	.67

a Anxiety, bipolar disorder, depression, psychoses, eating disorders, and postpartum disorders.

b Heart failure, congenital heart disease, and ischemic heart disease.

c End-stage renal disease and chronic renal failure.

d SUD: substance use disorder.

### Ninty-Day Inpatient Readmissions and ED Visits

These trends (ie, 30-day) persisted at 90-days postdischarge such that the enhanced outreach group experienced a 5.4% (95% CI 4.3%‐6.5%) and 3.8% (95% CI 2.5%‐5.0%) relative reduction in acute inpatient readmissions and ED visits, respectively, compared with the standard outreach group (both *P*<.001).

Secondary analyses revealed between-group differences across age, geographic region, geographic area, and comorbidities (Table 3). Similar to the 30-day results, members between the ages of 70 and 74 years experienced the greatest relative reduction in 90-day acute inpatient readmissions on the order of 8.5% (vs 2.2%‐5.9% for all other age groups). Members with behavioral health, cancer, diabetes mellitus type 1 or 2, heart conditions, low back pain, obesity, Parkinson disease, and renal-related conditions receiving enhanced messaging demonstrated greater reductions in acute inpatient readmissions at 90 days postdischarge (all *P*<.05) compared to members receiving standard messaging (Table 4).

**Table 4.** Relative change in 90-day acute inpatient readmissions and emergency department (ED) visits by demographic and clinical characteristics.

Characteristic	Change in 90-day inpatient readmissions (%), (95%CI)	*P* value	Change in 90-dayED visits (%), (95% CI)	*P* value
Age at admission (years)				
<65	–5.1 (–7.7 to –2.4)	<.001	–2.3 (–5.4 to 0.9)	.015
65‐69	–3.9 (–6.4 to –1.5)	.007	–1.4 (–4.2 to 1.5)	.27
70‐74	–8.5 (–10.7 to –6.3)	<.001	–8.9 (–11.5 to –6.3)	<.001
75‐79	–5.6 (–7.7 to –3.5)	<.001	–5.1 (–7.6 to –2.7)	<.001
80‐84	–5.9 (–8.2 to –3.7)	<.001	–0.7 (–3.2 to 1.8)	.56
>85	–2.2 (–4.2 to –0.2)	.09	–1.8 (–4.1 to 0.6)	.11
Region				
Midwest	–4.7 (–6.5 to –3.0)	<.001	–2.8 (–4.8 to –0.9)	<.001
Northeast	–8.5 (–10.1 to –6.9)	<.001	–5.3 (–7.2 to –3.3)	<.001
Southeast	–3.0 (–4.8 to –1.2)	.006	–3.3 (–5.5 to –1.0)	<.001
West	–2.6 (–5.1 to –0.1)	.10	–3.6 (–6.6 to –0.6)	.005
Geographic area				
Urban	–4.9 (–6.7 to –3.2)	<.001	–4.2 (–6.6 to –1.8)	<.001
Suburban	–9.9 (–11.9 to –8.0)	<.001	–6.1 (–8.5 to –3.7)	<.001
Rural	–3.2 (–4.4 to –1.9)	<.001	–2.7 (–4.1 to –1.3)	<.001
Comorbidities				
Behavior health^a^	–4.8 (–6.6 to –3.1)	<.001	–3.7 (–5.6 to –1.7)	<.001
Cancer	–4.4 (–6.2 to –2.7)	<.001	–4.4 (–6.5 to –2.3)	<.001
COVID-19	–0.5 (–4.6 to 3.7)	.85	–4.3 (–9.4 to 0.8)	.01
Dementia	–0.3 (–3.5 to 3.0)	.89	–3.5 (–7.6 to 0.6)	.02
Diabetes mellitus	–4.9 (–6.4 to –3.4)	<.001	–5.1 (–6.9 to –3.3)	<.001
Heart conditions^b^	–3.9 (–5.2 to –2.7)	<.001	–4.4 (–6.0 to –2.8)	<.001
Hepatitis	–3.7 (–9.7 to 2.3)	.24	–10.8 (–20.1 to –1.5)	<.001
HIV	–6.5 (–22.1 to 9.2)	.45	–31.7 (–54.6 to –8.8)	<.001
Low back pain	–6.4 (–8.0 to –4.7)	<.001	–6.3 (–8.2 to –4.4)	<.001
Multiple sclerosis	7.4 (–3.9 to 18.7)	.33	–0.6 (–14.8 to 13.5)	.92
Obesity	–4.5 (–6.4 to –2.7)	<.001	–5.3 (–7.5 to –3.0)	<.001
Parkinson disease	–8.5 (–15.8 to –1.3)	.04	–14.8 (–24.4 to –5.1)	<.001
Rheumatoid arthritis	–4.5 (–9.4 to 0.5)	.13	–5.1 (–11.2 to 0.9)	.04
Renal conditions^c^	–3.1 (–4.8 to –1.4)	.002	–2.6 (–4.8 to –0.4)	.004
SUD^d^	–2.9 (–6.2 to 0.3)	.11	–0.5 (–4.7 to 3.6)	.67

a Anxiety, bipolar disorder, depression, psychoses, eating disorders, and postpartum disorders.

b Heart failure, congenital heart disease, and ischemic heart disease.

c End-stage renal disease and chronic renal failure.

d SUD: substance use disorder.

## Discussion

### Principal Findings

This study sought to explore the impact of a payer-led, quality improvement initiative that was designed and implemented to deliver a low-intensity, multichannel educational outreach campaign to modify health behaviors with the goal of decreasing unplanned and avoidable hospital readmissions and ED visits at 30- and 90-days. Consistent with our hypothesis, members receiving the enhanced outreach campaign intervention experienced greater relative reductions in both outcomes compared with the standard outreach group at both 30- and 90-days postdischarge. Put into context, a 4% decrease in 30-day readmissions would translate to approximately US $600,000 in avoidable health care costs for every 1000 members or ≈US $136.5M for the included study sample of 227,470 members (assuming a conservative estimate of US $15,000 per readmission) [1].

The effectiveness of low-intensity, postdischarge follow-up has been examined in previous studies; however, a majority of research has focused on provider-led settings with direct clinician engagement and escalation mechanisms. Bressman et al [20] found that an automated, bi-directional, text-based follow-up intervention from their primary care physician following an index admission translated to 41% lower (adjusted odds ratio) 30-day acute inpatient readmissions or ED visits among 374 patients recently hospitalized (compared with a control, no messaging cohort). Similarly, Patel et al [21] were the first to evaluate the impact of provider-facing secure text messaging on patient outcomes among ≈6400 patients on inpatient services. Patients whose providers were engaged via text messaging to communicate patient information and to help facilitate medical decision-making demonstrated a significant decrease in length of stay on the order of ≈1 day. The larger effect size observed in these studies is likely attributed to differences in setting and intervention design. For example, their interventions were conducted within a single health care system, enabling direct provider engagement and follow-up, whereas this study implemented educational messaging at scale across a nationally distributed MA population. Additionally, the smaller sample size may have influenced effect size estimates by reducing the heterogeneity of outcomes.

This study expands upon the existing literature by evaluating the impact of a payer-led readmission avoidance campaign at scale. Despite the modest relative reductions observed in our study, the large-scale implementation across ≈368k members underscores the potential population-level impact of informatics-driven outreach. The success of the low-intensity multichannel educational outreach campaign is likely multifactorial. Access to extensive claims and clinical informatics data played a critical role in identifying high-risk patients in real-time, enabling the timely deployment of intervention efforts to reduce readmissions. Integrating these data into the outreach campaign enabled precision and effectiveness in reaching the target population. Unlike resource-intensive high-intensity interventions, the low-intensity campaign allowed for engagement with a larger number of members, ensuring that a broader population had equitable access to the necessary support and education to effectively manage their health at a critical moment that matters in their health care journey.

This multichannel outreach campaign embraced a holistic approach to care that was rooted in behavior change. Behavior change technique groupings (eg, goals and planning, shaping knowledge) [18], and intervention functions (eg, education, persuasion, and enablement) [19] were applied to modify health behaviors with the goal of reducing hospital readmissions. Numerous components that combined various self-management techniques, including knowledge acquisition, independent health monitoring, medication adherence, and lifestyle changes, comprehensively addressed member needs in a tailored manner. The multichannel aspect of outreach was designed to reinforce educational messaging that was personalized, contextual, and relevant. Additionally, targeted messaging and outreach efforts were deployed to both members and their providers on record, whenever possible. Specifically, campaign messaging encouraged members to proactively schedule postdischarge follow-up visits and communicate potential complications that would warrant additional clinical support. Simultaneously, payer-led provider alerts regarding their patients’ recent hospitalization equipped providers with pertinent information, enabling them to deliver more informed and timely interventions. Overall, this dyadic approach (ie, member-provider) aimed to bridge the communication gap and foster continuity of care by reinforcing the relationship between members and their existing care team.

Last, the low-intensity delivery of communications allowed for broader inclusion of Medicare-insured members across different regions of the United States compared with higher-intensity outreach, which is often cost and labor-intensive. Previous studies examining readmission reduction strategies have found that higher-intensity outreach combining multiple methods is more effective than only using one method, although these methods (eg, home visits, telephone calls, case management) are resultantly more costly compared with the outreach channels deployed in this study [22]. To the best of our knowledge, the study represents the first to investigate the effectiveness of combining multiple low-intensity outreach channels for this use case.

### Limitations

There are several limitations to this study. First, the study population is limited to Medicare Advantage members of one large national insurer in the United States with geographic concentrations in the Northeast and Midwest regions. A majority of members in this study self-report primary residence in rural areas (n=189,594 [51%]); notably higher than the average US population (14%) [23], which may limit generalizability to the broader US population. It is possible that differences in access to broadband internet and digital engagement tools may have influenced the effectiveness of electronic messaging, particularly among rural populations. However, the inclusion of multiple outreach channels, including direct mail and provider notifications, helped mitigate these potential disparities. It is difficult to systematically assess differences in access to technology by geographical location, as multichannel outreach was informed by member channel permissions. In addition, this study was not designed to examine differences in digital technology by geographic location. However, contemporary analysis (Feb 2025) suggests minimal differences in access and preference for digital outreach methods among Medicare Advantage members residing across rural, urban, and suburban locations, with 68%‐69% of members permitting SMS, 75%‐78% permitting email, and 92%‐93% permitting IVR contact across all regions. Interestingly, email open rates (2024 data) indicate slightly lower engagement among rural members (60.5%, *P*<.0001) compared with suburban (64.3%) and urban (62.8%), which may signal potential differences in digital engagement and warrant additional exploration. Nevertheless, this study presents outcomes for a population often underrepresented in the literature and advances our understanding of potential disparities in hospital readmissions.

Second, this study infers readmission and ED visit reductions to be driven by the campaign interventions and does not consider additional (ie, not plan sponsored) interventions. Third, the time horizon may not fully capture the impact of the COVID-19 pandemic on members’ behaviors, which continues to evolve. Base rates are unable to be reported due to commercial data use agreements; however, these findings from this large-scale intervention provide valuable insights for designing and implementing future readmission reduction strategies across diverse health care settings. Finally, the possibility always exists that unmeasured confounding variables and potential selection bias may influence outcomes.

Despite the recognized limitations, this study possesses several key strengths. This study represents a retrospective evaluation of a rigorously designed and pragmatic quality improvement intervention. The included study population consisted of a large sample size of 368,693 Medicare Advantage-insured, geographically diverse, older adults across the nation. By focusing on Medicare Advantage beneficiaries, the study addresses a critical population with unique health care needs, contributing to the development of effective strategies to reduce readmissions and improve member and patient outcomes in the population most likely to have gaps in care. The use of claims data enriches the results, providing real-world evidence of outcomes. The integration of additional member data, including plan benefit structure and overlapping programs, enhances the study’s precision and effectiveness in minimizing confounding bias. Finally, the campaign’s grounded approach in evidence-based practice and health behavior theoretical constructs reinforces the robustness of the intervention and its potential applicability to the generalized population.

### Future Research

There are several opportunities for additional exploration. Immediate future research will aim to explore the incremental effect of additional channels. Interestingly, post hoc analysis within the enhanced outreach group revealed that members who received more channels experienced fewer ED admissions than those receiving fewer channels. Specifically, members who received 4 outreach channels had fewer 30-day acute inpatient readmissions and ED visits than members receiving campaigns via one outreach channel, indicating an additive effect of incremental channels (*P*<.001). While interesting, we caution that these results are preliminary and warrant additional evaluation in a controlled, hypothesis-driven study.

In addition, investigating the impact of low-intensity channels based on specific sub-populations, diseases or conditions, and health care settings can provide targeted insights for tailored interventions. As previously mentioned, incorporating more granular data on channel engagement by geographic location could potentially refine multichannel strategies to optimize outreach effectiveness, particularly in rural populations. Future explorations should explore whether reliance on postcharge digital messaging could unintentionally widen the “digital divide” or disparities in access to care. Additionally, exploring best practices in human-centered design that increase member satisfaction, engagement, and resultant clinical and economic outcomes will further optimize and elucidate campaign effectiveness.

### Conclusions

Payer-led, personalized educational messaging using multiple low-intensity channels of delivery, including digital health communications such as text message, email, and IVR, can effectively reduce inpatient readmissions and ED visits within 30- and 90-days of discharge among the Medicare Advantage member population.

## Supplementary material

10.2196/63841 Multimedia Appendix 1 Screenshot displaying enhanced preadmission messaging sent to members via direct mail channel prior to an upcoming hospital stay. Preadmission outreach content focused on priority actions to take in advance of a hospital stay, with emphasis on preparation for the eventual return home (eg, nutritious meal planning, removal of fall risk hazards), scheduling follow-up visits prior to admission, and the importance of medication filling and adherence).

10.2196/63841 Multimedia Appendix 2 Screenshot displaying enhanced postdischarge messaging sent to members via direct mail following hospital discharge to home. Postdischarge outreach content focused on a member’s individual recovery journey by providing a “recovery tracker” calendar for personalized tracking of pain levels, upcoming appointments, and any other key notes to share with their care team at their next follow-up visit. The content reinforced the importance of going to follow-up visits, filling prescriptions and taking them as directed, as well as common warning signs suggestive of a potential complication that would warrant care.

## Abbreviations

ED: emergency department
HRRP: Hospital Readmission Reduction Program
IVR: interactive voice response
