# Supplementary figures and images for: An Informatics-Based, Payer-Led, Low-Intensity Multichannel Educational Campaign Designed to Decrease Postdischarge Utilization for Medicare Advantage Members: Retrospective Evaluation
Source: JMIR Hum Factors. 2025 May 27;12:e63841. doi: 10.2196/63841 (PMC12133070; doi:10.2196/63841)

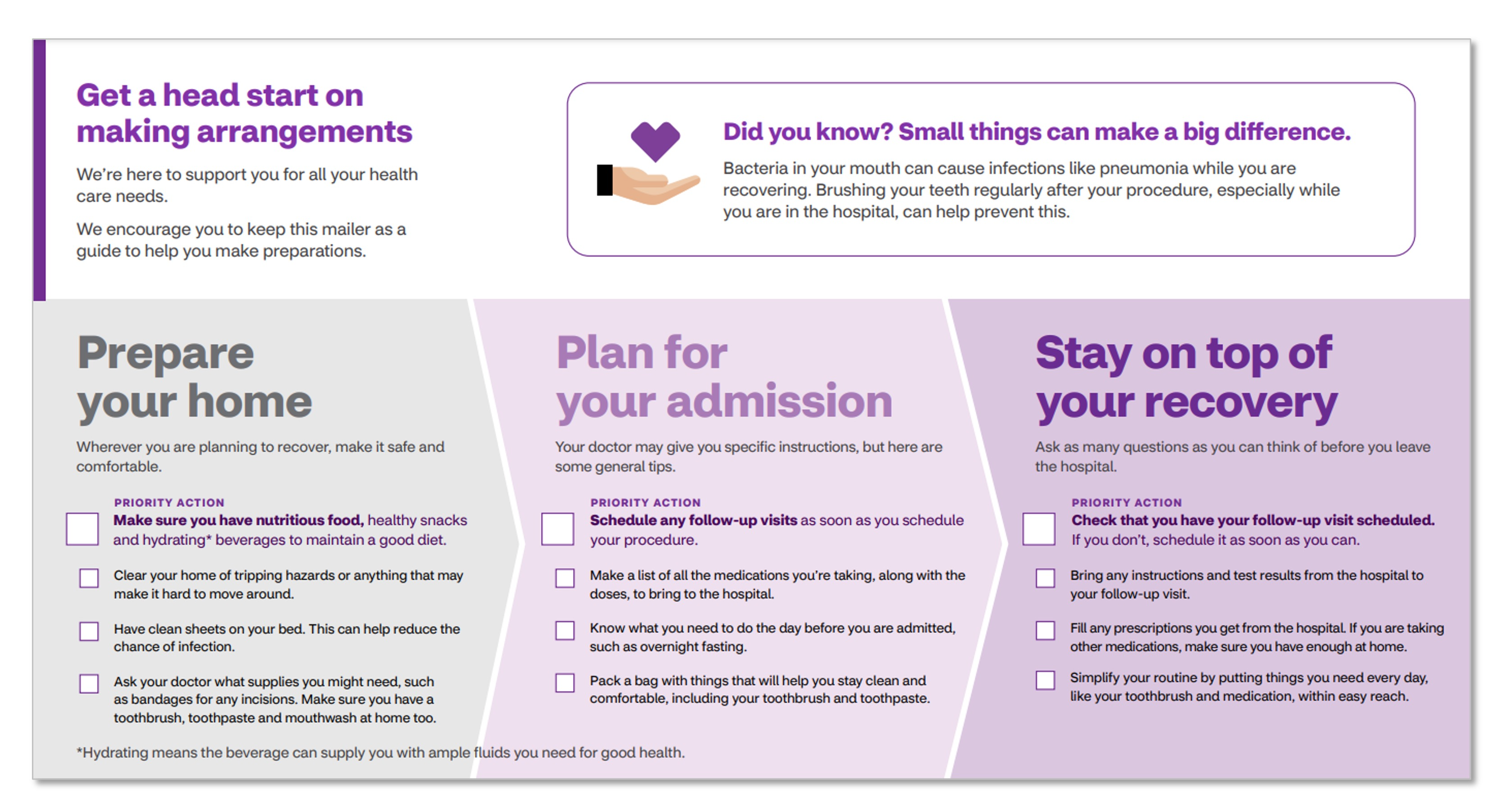

Supplement: Multimedia Appendix 1 [file humanfactors-v12-e63841-s001.png]

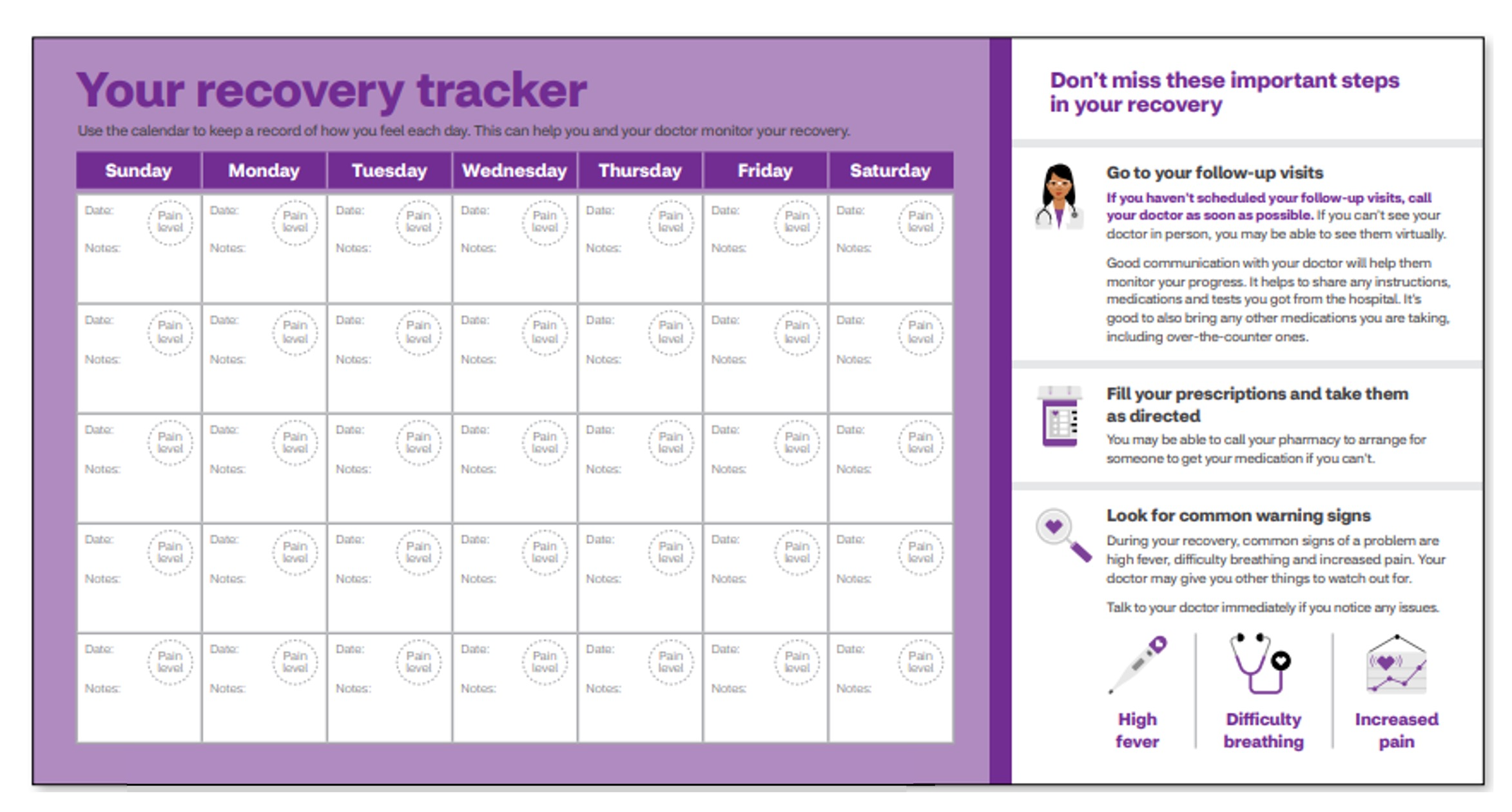

Supplement: Multimedia Appendix 2 [file humanfactors-v12-e63841-s002.png]
